# Spawning Performance and Sex Steroid Levels in Female Pikeperch *Sander lucioperca* Treated with Poly(lactic-co-glycolic acid) Microparticles

**DOI:** 10.3390/ani12020208

**Published:** 2022-01-16

**Authors:** Jindřiška Knowles, Jakub Vysloužil, Tomáš Policar, Sylvain Milla, Martina Holická, Peter Podhorec

**Affiliations:** 1 Faculty of Fisheries and Protection of Waters, South Bohemian Research Center of Aquaculture and Biodiversity of Hydrocenoses, University of South Bohemia in České Budějovice, Zátiší 728/II, 38925 Vodňany, Czech Republic; policar@frov.jcu.cz (T.P.); podhorec.peter@seznam.cz (P.P.); 2 Department of Pharmaceutical Technology, Faculty of Pharmacy, Masaryk University, Palackého třída 1946/1, 61200 Brno, Czech Republic; vyslouzilj@pharm.muni.cz (J.V.); 93holicka.martina@seznam.cz (M.H.); 3 Unit of Animal Research and Functionalities of Animal Products, University of Lorraine, INRA 340, 54506 Vandoeuvre-l`es-Nancy, France; sylvain.milla@yahoo.com

**Keywords:** aquaculture, GnRHa, induced ovulation, reproductive dysfunction, sustained drug release

## Abstract

**Simple Summary:**

Pikeperch *Sander lucioperca* is a promising candidate for intensive aquaculture. However, controlled reproduction has become the major bottleneck in pikeperch production. To improve and optimize its artificial reproduction, an effective method for hormone treatment is needed. The use of a poly(lactic-co-glycolic acid) microparticle-sustained-release system to administer gonadotropin-releasing hormone agonist to pikeperch resulted in acceptable reproductive output. Our results establish the potential of poly(lactic-co-glycolic acid) microparticle as a novel tool for hormone treatment in fish.

**Abstract:**

Pikeperch *Sander lucioperca* is a piscivorous species considered a promising candidate for the diversification of intensive aquaculture. This study aimed to determine the effect of a sustained-release delivery system incorporating mammalian gonadotropin-releasing hormone agonist (mGnRHa) into poly(lactic-co-glycolic acid) (PLGA) microparticles on the sex steroid levels and aspects of artificial reproduction of pikeperch. Fish were divided into four groups and injected with 20 µg mGnRHa/kg, 5-day release microparticles encapsulated with 5 µg GnRHa/kg BW (PLGA 5), 20 µg GnRHa/kg (PLGA 20), or 1 mL/kg 0.9% NaCl (control). Cumulative percentage ovulation was 100% in the PLGA 5 group, significantly higher than in other tested groups. No differences among groups were observed in latency or fecundity. The level of 11-ketotestosterone (11-KT) peaked at 40 h post-injection, and was sustained during ovulation, in all treated groups. The 17β-estradiol (E2) concentration increased in the mGnRHa-only group immediately after hormone injection, while both PLGA groups showed a reduction in E2 after injection, continuing to decrease until ovulation. A low dose of mGnRHa in PLGA microparticles significantly improves induction of ovulation and results in acceptable reproductive performance, which may positively affect pikeperch production under controlled conditions.

## 1. Introduction

Pikeperch *Sander lucioperca* is the most valuable freshwater fish species for European intensive aquaculture due to its exquisite meat quality, good growth performance, and high market demand [1]. Like other Percidae, pikeperch is an annual spawner with group synchronous ovarian development [2]. Spawning usually occurs from March through May when water temperature is 10–16 °C [3]. The controlled reproduction of pikeperch has long challenged aquaculturists, with initial attempts to obtain gametes of the species reported in 1928 [4]. To date, the methods usually used to accomplish this are natural spawning [5] or support of spawning by placing artificial nests (synthetic turf, brushes, coconut mats) inside rearing tanks [6,7,8]. These methods have the advantages of minimal handling and labor, but are often associated with low fertilization and with loss of eggs due to spawning outside the nest [8,9]. The techniques also do not allow the controlled reproduction of individual fish, often required for breeding programs or triploidisation [10], necessitating stripping for in vitro fertilization. Although it shows advantages, artificial reproduction followed by in vitro fertilization is a challenging process complicated by difficulty in timing of stripping, spontaneous eggs release, post-ovulatory ageing, high broodstock mortality, and variable egg quality [8,11].

Fish artificial reproduction aided by hormone stimulation dates to the early 20th century. Hormonal induction of ovulation in fish involves use of either gonadotropins or a gonadotropin-releasing hormone analogue (GnRHa) in a liquid or sustained-release delivery system [12]. In pikeperch, the commonly used preparations for induction of ovulation are human chorionic gonadotropin (hCG) and GnRHa [8,9]. In pikeperch, hCG treatment has a negative effect on egg quality [11] and can enhance cortisol production [13] that may further impact egg quality [14].

In addition to the hormone used, the method of administration is an important factor influencing treatment efficacy [15]. The development of an advanced drug delivery system opens new possibilities for avoiding multiple injections and enabling the release of bound hormones at a desired dose for a predetermined period of time [16]. In the past 20 years, a variety of sustained GnRHa delivery systems have been successfully tested in induction of ovulation and spermiation in fish. Among them, copolymer of ethylene [17] and cholesterol pellets [18] have been the most used in induction of reproduction in fish. Nevertheless, it is important to highlight that these delivery systems are used to induce ovulation and spermiation particularly in marine fish and salmonids [17]. Several more sustained drug delivery systems, such as Freund’s incomplete adjuvant [19], microspheres of copolymer of fatty acid dimer, sebacic acid, lactide-glycolide [20], and chitosan-gold nanoconjugates [21], have been tested in fish. However, these systems did not gain wider commercial use in the treatment of freshwater fish reproductive dysfunctions. Among drug delivery systems used in veterinary and human medicine, the poly(lactic-co-glycolic acid) (PLGA) microparticles have emerged as one of the most promising matrixes for binding drugs, due to its simple preparation [22], biocompatibility, biodegradability [23], and its facility of encapsulating a wide variety of active substance [24,25,26]. 

The aim of this study was to determine the effect of hormone treatment using mammalian gonadotropin-releasing hormone agonist (mGnRHa) incorporated into PLGA microparticles in pikeperch females and compare its efficacy with that of mGnRHa treatment alone and with physiological saline solution.

## 2. Materials and Methods

### 2.1. Experimental Groups and Design

Pikeperch females (*n* = 40; 818 ± 295 g) were collected from earthen ponds in South Czechia in late March and transported to the experimental facility of the Faculty of Fisheries and Protection of Waters in Vodnany (49°N, 14°E) where they were held in storage ponds and fed forage fish *Pseudorasbora parva* until the spawning season in April. Oocyte maturity stage was determined in each female according to described methods [10]. Oocytes were collected from the urogenital papilla via a polyethylene cannula (2.7 mm) and the sampled oocytes were treated with a clearing solution (ethanol:formalin:glacial acetic acid in the ratio of 6:3:1). Females at oocyte maturation stage III were randomly divided into four groups of 10 and intramuscularly injected under the dorsal fin according to the following protocols:

- 0.9% NaCl: saline solution only (Braun Melsungen AG, Melsungen, Germany), single injection of 0.9% NaCl at 1 mL/kg body weight (BW).
- mGnRHa: single injection of Supergestran^®^ (Nordic Pharma, Jesenice, Czech Republic) at 20 µg/kg BW [mGnRHa (D-Tle^6^, Pro^9^, NEt-mGnRHa)].
- PLGA 5: 5-day release microparticles with encapsulated GnRHa ([D-Ala^6^, des-Gly^10^]GnRH-ethylamide) (APExBIO, Houston, TX, USA) at 5 µg/kg BW = GnRHa at 1 µg/kg BW per day.
- PLGA 20: 5-day release microparticles with encapsulated GnRHa ([D-Ala^6^, des-Gly^10^]GnRH-ethylamide) (APExBIO, Houston, TX, USA) at 20 µg/kg BW GnRHa = 4 µg/kg BW per day.

**Figure 1:**
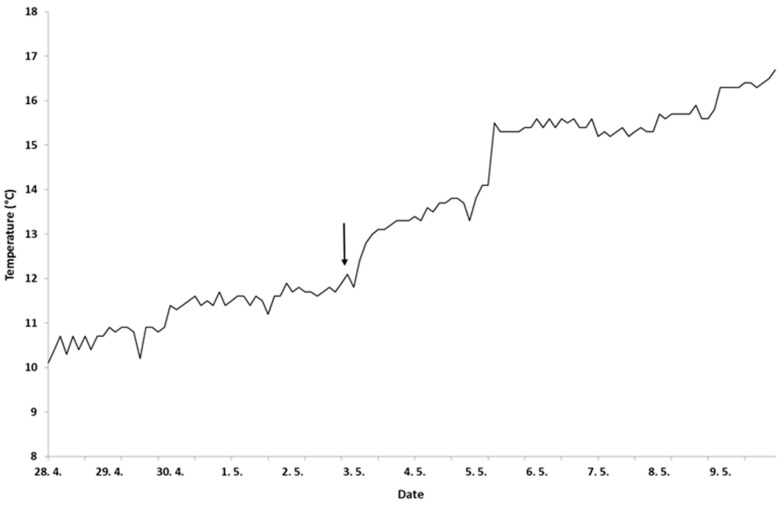
Water temperature over the course of 12 days auto-recorded by data logger (Lascar Electronics, EL-USB-1-RCG) at 1-h intervals. Arrow indicates the date of hormone injection.

The choice of doses used in the present study were based on the findings of various studies. A single injection of dose 5 µg GnRHa/kg BW have not proven being successful in formerly published studies and thus this group was not included in the experiment [27,28]. Following injection, each group was kept in a 1 m^3^ flow-through tank under ambient water temperature. Water temperature was measured hourly by an auto-recording data logger (EL-USB-1-RCG, Lascar Electronics, Whiteparish, UK) (Figure 1). The oxygen saturation (%) and pH were measured daily at 06:30 and 14:00 h with a combined pH and oxygen meter (MultiLine P4, WTW, Weilheim, Germany). Water quality parameters were pH 7.2 ± 0.2, oxygen saturation 80.4 ± 9.1%, flow rate 12.5 L/min, temperature 15.1 ± 0.96 °C, ammonia concentration, <0.02 mg/L; nitrite, <0.02 mg/L; and photoperiod 14L:10D with light intensity of 30 lx.

Blood (2 mL) was collected by caudal venipuncture into 5 mL heparinized syringes from each fish at 0, 20, 40, and 168 h post-stimulation for steroid level analysis. Plasma samples were obtained by centrifugation at 1500× *g* for 10 min at 10 °C (Eppendorf 5427 R, Eppendorf, Hamburg, Germany) and immediately frozen and stored at −80 °C until analyses.

### 2.2. Microparticle Formation

Microparticle preparation used a standard water-in-oil-in-water combination (w1/o/w2). Ten mg of alarelin acetate (APExBIO, Houston, TX, USA) was dissolved in 1.5 g of warmed (50 °C) 9.1% gelatine solution (w1). PLGA RESOMER^®^ RG 753H (800 mg) (Evonik, Darmstadt, Germany) was dissolved in 5 mL dichloromethane (Penta, Prague, Czech Republic) (oil phase). The w1 and oil phases were pre-mixed by vortexing for 30 s and homogenized for 60 s (Ultra-Turrax T25, Ika Werke, Staufen Im Bresgau, Germany). The resulting w1/o emulsion was pre-mixed for 60 s on a homogenizer with 12 g of warmed (50 °C) 1% poly(vinyl alcohol) solution (PVA, Mw 31,000–50,000, 98–99% hydrolyzed; Sigma Aldrich, St. Louis, MO, USA) to create a concentrated double emulsion w1/o/w2. The concentrated emulsion was immediately poured into 200 g of 0.1% PVA/2.0% NaCl. The resulting w1/o/w2 emulsion was stirred for 2 h to completely evaporate dichloromethane. Particles were collected by centrifugation, re-suspended in purified water, and lyophilized.

**Figure 2:**
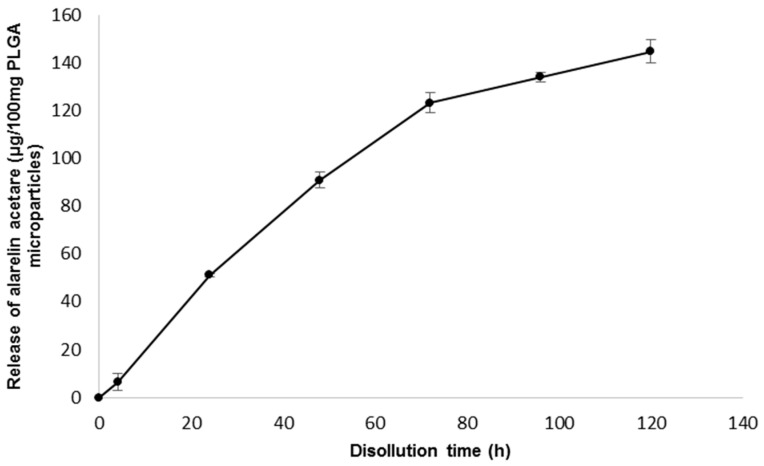
Dissolution profile of alarelin acetate release from poly(lactic-co-glycolic acid) microparticles.

To provide information about drug release from the PLGA microparticles, an in vitro dissolution test was carried out in 10 mL glass vials at 15.0 °C ± 0.5 °C using 100 mg PLGA microparticles with alarelin acetate embedded in 0.4 mL 1% agar gel. After the gel solidified, an additional 0.8 mL agar gel was applied as a cover layer. After solidification, 5 mL of pH 7.0 phosphate buffer was added. Samples were taken for seven days. The entire volume of dissolution medium was removed and replaced with fresh buffer. The dissolution was performed in triplicate. Determination of alarelin acetate in dissolution medium was performed by HPLC (Agilent 1100, Agilent Technologies, Santa Clara, CA, USA) on a Nucleodur 10-5 CN-RP column (Machery-Nagel, Duren, Germany) with acetonitrile: 20 mM H_3_PO_4_ (16:84 *v*/*v*) at a flow rate of 0.8 mL/min and spectrophotometric detection at 220 nm (Figure 2).

### 2.3. Stripping of Broodfish

Beginning 48 h post-hormone treatment, the fish were examined hourly by a gentle abdominal massage. Those showing signs of ovulation were immediately transferred to a clove oil water bath (0.03 mL/L), and eggs were stripped by gentle abdominal massage under dry conditions into a dry bowl. Eggs were weighed using a balance (PCB 1000-2, Kern, Germany with accuracy of 0.01 g) separately for each female. Three samples of ~1 g per spawning were randomly selected and weighed using a balance (ALJ 220-4, Kern, Balingen, Germany) with accuracy of 0.0001 g and counted for determination of absolute fecundity (total number of eggs per female) and relative fecundity (total number of eggs per kg BW). 

After fertilization according to a previously described method [28], three samples of ~100 eggs were counted and incubated in separate incubators in a recirculating system at 16 ± 0.5 °C as described by Blecha et al. [6]. Hatching began on the fifth day post-spawning and the freshly hatched free-swimming larvae were counted. The hatching rate was determined as follows:

Hatching rate = (NL/NE) × 100(1)

where NL is the number of hatched larvae and NE is the total number of eggs stocked in the incubator.

### 2.4. Enzyme-Linked Immunosorbent Assay (ELISA)

Plasma levels of testosterone (T), 11-ketotestosterone (11-KT), and 17β-estradiol (E2) were quantified by ELISA, and each standard and plasma sample was run in duplicate.

Concentration of T (KAPD1559) and E2 (KAP0621) were analyzed by ELISA using commercially available kits (DIAsource, Ottignies-Louvain-la-Neuve, Belgium) according to the manufacturer’s instructions. The androgen 11-KT was assayed with the Cayman 11-KT EIA kit (582751, Cayman Chemical, Ann Arbour, MI USA). The intra-assay coefficients of variation (calculated from the duplicate sample) were less than 7% in all tests and inter-assay coefficients of variation were less than 6.5%. Absorbance was read with a microplate plate reader (PlateReader AF2200, Eppendorf, Hamburg, Germany).

### 2.5. Data Analysis

Data are presented as mean ± standard error of the mean (SEM). Statistical analysis was conducted with Statistica v. 12 CZ (StatSoft, Tulsa, OK USA). Normality and homogeneity (Cochran C) of data were tested on raw data, percentage data were arcsin transformed, and steroid data log transformed to satisfy homogeneity of variance requirements. Significant differences were analyzed by one-way ANOVA. If significant differences were found by ANOVA, Tukey, or HSD test were applied for detailed multicomparison assay. Ovulation rate was analyzed by the χ2 analysis. For all tests, the level of significance was set at *p* < 0.05.

## 3. Results

Ovulation rate (number of ovulated females during the experiment) was 100% in the group treated with 5 µg GnRHa/kg BW (PLGA 5) and was significantly higher than rates obtained in other groups. Cumulative ovulation in groups treated with mGnRHa only and with PLGA 20 was 40%, while no ovulation was seen in the saline-only control group. The latency period (time from injection to ovulation) ranged from 80 to 128 h post-hormone treatment with no significant differences among treated groups. No significant differences were found among groups in relative or absolute fecundity, and high inter-individual variation was recorded. High SEM of both measures of fecundity reflected the wide range in egg numbers obtained from individual females. Hatching rate was 53.4–68.0% in the successfully spawning groups with no significant differences among groups (Table 1).

**Figure 3:**
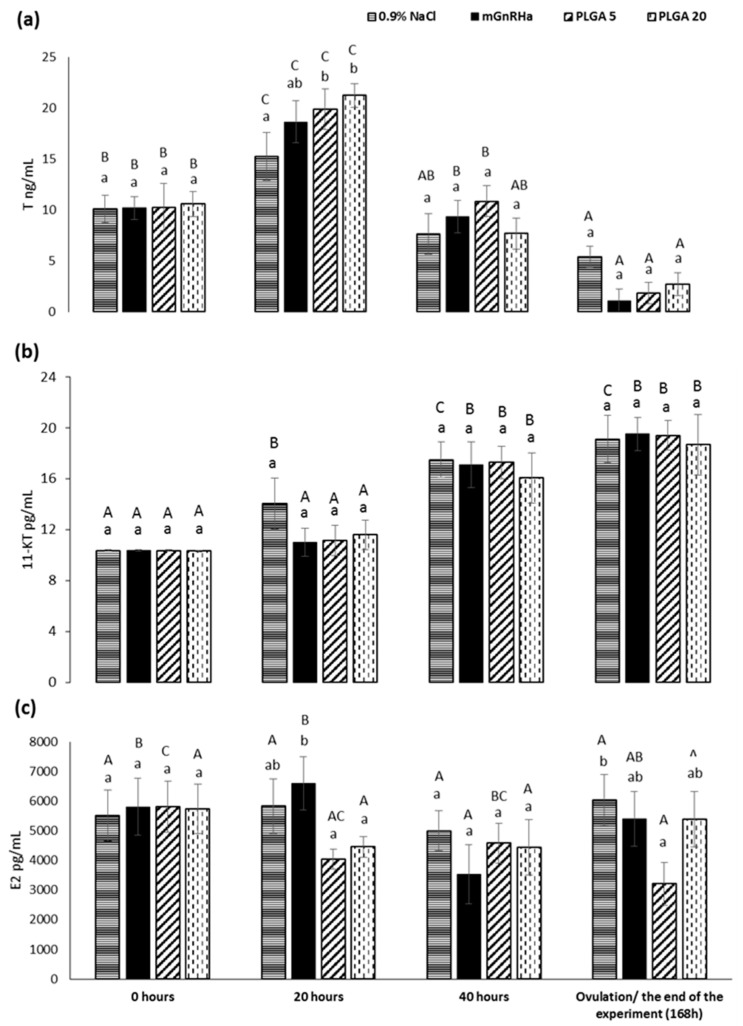
Changes in plasma levels of testosterone (**a**), 11-ketotestosterone (**b**), and 17β-estradiol (**c**) in groups injected with 0.9% NaCl at 1 mL/kg; single injection of mammalian gonadotropin-releasing hormone agonist (mGnRHa) at 20 µg/kg; 5 µg mGnRHa/kg BW embedded in PLGA microparticles (PLGA 5); and 20 µg mGnRHa/kg BW embedded in PLGA microparticles (PLGA 20). ^a,b^ Significant differences among groups at a sampling point are indicated by lower case letters (one-way ANOVA). ^A,B,C^ Significant differences within an experimental group are indicated by upper case letters (one-way ANOVA).

The level of plasma T increased in the PLGA groups 20 h after hormone stimulation compared to the control group but declined precipitously at ovulation. 11-KT showed an increasing trend from 40 h post-hormone treatment in all groups with highest level at ovulation time. No significant effect was reported with either androgen. Plasma E2 level increased in the mGnRHa group immediately upon hormone administration compared to the PLGA groups. However, such inter-group difference was not detectable after 40 h (Figure 3).

## 4. Discussion

The present study was carried out to develop and investigate the efficacy of microparticle-sustained delivery of mGnRHa. The use of PLGA microparticles at 5 µg/kg BW induced 100% ovulation, significantly higher than in mGnRHa group (40%).

High effectiveness of low mGnRHa dose incorporated in PLGA is an interesting result because higher acute doses of GnRHa (up to 100 µg/kg) are usually used to induced ovulation in pikeperch [27,28]. Encapsulation of GnRHa increases its stability [29], which otherwise has a short lifetime in blood [30]. Sustained release of mGnRHa shows advantages compared to conventional forms, containing controlled release, low toxicity, better efficacy and avoids the necessity of using multiple injections [16]. The high response of pikeperch to sustained mGnRHa release from PLGA microparticles compares well with studies of systems for prolonged GnRHa release in other fish species. A positive effect of sustained mGnRHa release on fish reproduction has been confirmed common carp *Cyprinus carpio* [21], common snook *Centropomus undecimalis* [31], spotted rose snapper *Lutjanus guttatus* [32], greater amberjack *Seriola dumeril* [33] Atlantic cod *Gadus morhua* [34], starry flounder *Platichthys stellatus* [35] and many more [17]. However, sustained GnRHa release systems are commonly used in marine fish species and some salmonids [17] but are understudied in freshwater fish species. We confirm that this route of hormone administration is beneficial to further progress in managing reproduction of freshwater fish species.

Production of eggs of inconsistent quality [36] is a major bottleneck in artificial reproduction of pikeperch [14]. Effectivity to induce ovulation in pikeperch by a single injection of mGnRHa is significantly outperformed by two consecutive mGnRHa injections [37,38]. Nevertheless, a single injection is preferable to reduce the stress connected with hormone treatment and manipulation, which may be associated with poorer egg quality [15]. In some species, higher GnRHa dosages can lead to low ovulation rate [39,40] and additionally cause production of low-quality eggs [41,42]. Our obtained hatching rate of 53.4–68.0% for all groups was not significantly affected by hormone treatment. Similar hatching rate (51–71%) in pikeperch was confirmed in semi-artificial propagation after stimulation with hCG [7] as well as in an experiment focused on out-of-season pikeperch spawning (49–64%) [43]. Our results, showing no difference among groups, indicate that the route of mGnRHa administration affects the ovulatory event but not egg quality. Other researchers also reported no differences in fertilization and hatching rate, and thus conclude that the long-term elevated LH levels induced caused by sustained GnRHa delivery does not adversely affect the quality of eggs [44,45]. A positive influence of sustained GnRHa release on the number of eggs was confirmed in some fish species, such as red porgy *Pagrus pagru* [46], common carp [21], turbot *Scophthalmus maximus* [47], and yellowtail flounder *Pleuronectes ferrugineus* [48]. Nevertheless, no differences were observed in terms of absolute and relative fecundity in our study. The same findings were confirmed in other studies, where no effect of various hormonal treatments and dosages on the pikeperch fecundity was noticed [28,38].

Latency period was not significantly modified by PLGA hormonal treatments compared to mGnRHa group and was in the range of 94.6 ± 13 h. Similar latency was reported in female pikeperch after a single injection of mGnRHa [28]. Shorter latency is often observed in females treated with gonadotropins acting directly on the gonads (carp pituitary extract, hCG) compared to treatments acting on the brain (GnRHa) [12]. Studies to assess modulation of the latency period in percids via administration of certain spawning agents have shown negative results [8,14,49].

To improve artificial reproduction of pikeperch, it is crucial to understand the physiological changes during the final maturation in cultured fish [13]. Decrease of reproductive capacity in domesticated pikeperch [50] is due to depression of sex steroids during oogenesis. Lower levels of E2 in domesticated pikeperch females may be due to its ineffective conversion from T compared to that in wild females [51,52]. In fish with synchronous ovarian development, the concentration of E2 increases during vitellogenesis while T increases later, and both levels drop during final oocyte maturation and ovulation [53]. This trend was confirmed in our experiment in females stimulated with mGnRHa. In the groups treated with PLGA microparticles, the level of E2 showed a non-significant downward trend with no increasing levels observed. As E2 is known to inhibit the entry of oocytes into the final oocyte maturation in a range of fish species, we assume that lower E2 after PGLA injection might be linked to the increase of ovulation, at least with the dose of 5 µg GnRHa/kg BW.

In the present study, plasma T levels showed significant increasing values after 20 h in the groups stimulated with PLGA microparticles compared to the control group, indicating stimulation of gonadotropin-mediated sex steroid secretion by the gonad. The hormone treatment significantly stimulated production of T, which decreased toward ovulation. This trend has been reported in other studies of cultured pikeperch [14]. The results of our study show that hormone stimulation, irrespective of administration method, is effective in stimulation of T secretion in pikeperch. As E2 can be converted directly from T [51], our findings suggests that the lower and unstable concentrations of E2 may be caused by insufficient conversion from T.

The level of 11-KT increased after 40 h in all groups regardless of treatment. The role of 11-KT in vitellogenesis and ovulation is scarcely studied, and further investigation is needed. We found higher levels of 11-KT in the end of the experiment in female pikeperch, which may indicate an effect on ovulation [54]. Whether it is T or 11-KT, the absence of and effect of treatment type on androgen regulation suggests that the route of GnRH exposure does not influence the androgen secretion and that the improvement of the ovulation rate with PGLA 5 is not due to differential release.

The PLGA microparticles released GnRHa for up to 5 days at temperature 15 °C. Hormonal treatment employing PLGA microparticles may have important benefits over acute hormone injections or solid implants. In contrast with cholesterol [17] and EVAc implants [44], the PLGA polymer is biodegradable and breaks down into lactic and glycolic acid, which enter the Krebs cycle. Following that, it is further decomposed to water and carbon dioxide [54], which can be an important factor in broodstock that may be further sold on the market. Another advantage over solid implants is that PLGA microparticles dose can be simply adjusted, which allows the treatment of species from 20 kg to 20 g [20]. Moreover, due to the prolonged GnRHa release from the PLGA microparticles, the necessity of using re-injections and repetitive handling can be eliminated [16]. It is important to note that the tank condition may affect fish reproduction. This was not reflected in the methodology of the study and should not be omitted in future studies. Future studies should give special importance in using a control group injected with pure PLGA microparticles as well as the immune response of the fish to treatment. Further investigation of efficacy of PLGA microparticles in inducing ovulation and spermiation, gametes quality, and the steroid feedback will be studied in various fish species.

## 5. Conclusions

A low dose of mGnRHa in PLGA microparticles is effective in inducing ovulation. The sustained release of GnRHa encapsulated in PLGA microparticles results in acceptable reproductive output, establishing its potential as a tool for the induction of ovulation in pikeperch. The ease of producing the microparticles and potential for controlled GnRHa release may be effective in overcoming reproductive dysfunction in cultured fish.

## Figures and Tables

**Table 1:** Characteristics of ovulation in pikeperch relative to mGnRHa delivery protocol. Fish were injected with 0.9% NaCl at 1 mL/kg; single injection of mGnRHa at 20 µg/kg; 5 µg GnRHa/kg BW embedded in PLGA microparticles (PLGA 5); and 20 µg GnRHa/kg BW embedded in PLGA microparticles (PLGA 20).

Group	Injected/Ovulated	Latency Period (h)	Absolute Fecundity (Eggs/Female)	Relative Fecundity (eggs/kg BW)	Hatching Rate (%)
0.9% NaCl	10/0 ^a^				
mGnRHa	10/4 ^a^	84.5 ± 0.65	85,094 ± 39,731	83,943 ± 36,100	57.7 ± 7.9
PLGA 5	10/10 ^b^	96.8 ± 1.03	109,781 ± 24,872	128,184 ± 27,811	68.0 ± 4.4
PLGA 20	10/4 ^a^	90.5 ± 1.95	61,784 ± 41,289	52,784 ± 31,868	53.4 ± 5.1

^a,b^ Different superscripts within a column indicate significant difference (*p* < 0.05).

## Data Availability

The data presented in this study are available on request from the corresponding author.
